# Alcohol Consumption, Beverage Preference, and Diet in Middle-Aged Men from the STANISLAS Study

**DOI:** 10.1155/2012/987243

**Published:** 2012-09-29

**Authors:** Bernard Herbeth, Anastasia Samara, Maria Stathopoulou, Gérard Siest, Sophie Visvikis-Siest

**Affiliations:** EA 4373, Génétique Cardiovasculaire, Université de Lorraine, Nancy 54000, France

## Abstract

The question about differences in dietary patterns associated with beer, wine, and spirits is still unresolved. We used diet data from 423 middle-aged males of the STANISLAS Study. Using adjusted values for covariates, we observed a negative significant association between increasing alcohol intakes and the consumption of milk, yogurt, and fresh/uncured cheese, sugar and confectionery, vegetables and fruits, and a significant positive relationship with cheese, meat and organs, pork-butcher's meat, and potatoes. In addition, the first dietary pattern identified by factor analysis (characterized a more prudent diet) was inversely related to alcohol intakes. Conversely, when analyzing daily consumption of specific food groups and diet patterns according to beverage preference (wine, beer, and spirits), no significant difference was observed. In conclusion, in this sample of middle-aged French males, there was a linear trend between increasing alcohol intakes and worsening of quality of diet, while no difference was observed according to beverage preference.

## 1. Introduction

Alcohol is linked to an extensively documented J-shaped dose-effect curve, with light to moderate consumption reducing cardiovascular and overall mortality, whereas excessive drinking has the opposite effect [1–3]. Moreover, drinking pattern (heavy episodic or binge drinking versus a steady pattern of consumption [4, 5]), type of alcoholic beverage (wine, beer, spirits), and various related lifestyle and sociocultural factors may account for differences in health benefits or adverse effects associated with alcohol drinking. In the field of nutrition, prior research supports that increasing levels of alcohol consumption are associated with poorer dietary patterns [6–9]. Generally, increased alcohol intake was associated with higher consumption of potatoes and animal products such as meat, meat products, and pork-butcher's meat and low consumption of dairy products such as milk, yogurt, and fresh/uncured cheese, fruits and vegetables, and pastries and cookies. In addition, variation in diet associated with the preferred drink may explain why a type of alcoholic beverage seems to have specific effects on ischemic heart disease mortality. Some studies in Danish or American populations found that wine drinkers tend to have a healthier lifestyle profile (including diet) than beer or spirit drinkers [10–12]. However, in Navarra (Spain) dietary patterns between wine, beer, or spirit drinkers did not significantly differ [13]. Even within the same country, geographical factors could play an important role; for example, in France, living area, diet behaviors, and alcoholic beverage preference were strongly associated in the study of Ruidavets et al. [9].

Since data about French are limited, this present study aims to describe the associations of alcohol consumption and alcoholic-beverage preferences with dietary patterns measured in term of food groups and pattern in 423 middle-aged males living in Eastern France.

## 2. Material and Methods

### 2.1. Subjects

This work is based on the STANISLAS Family Study, a 10-year longitudinal study conducted since 1994 on 1,006 families selected at the Center for Preventive Medicine of Vandoeuvre-lès-Nancy (east of France) [14, 15]. These families (2 parents and at least 2 children between 10 and 26 years) were identified from the files of the State Health Insurance Fund and invited every 5 years for checkups at the Center for Preventive Medicine. Due to the design of the STANISLAS Family study, subjects were of French origin and were free from acute or chronic diseases such as stroke, myocardial infarction, or cancer. At baseline, a random subsample of these families (about 45%) had to complete a 3-day food-intake diary. We performed this cross-sectional analysis on data of the entrance checkup (1994-95) from the sample of 423 fathers, who completed the 3-day food-intake diary and who had available covariate measurements (aged: 30–60 years, median age: 42 years, alcohol intakes: 0 and 112 g/day). Each subject gave written informed consent for participating in this study, which was approved by the “Comité Consultatif de Protection des Personnes dans la Recherche Biomédicale de Lorraine” (France). In addition, we certify that all applicable governmental regulations concerning the ethical use of human volunteers were followed during this research.

### 2.2. Dietary Assessment and Data Collection

Dietary intake was assessed with a 3D dietary record, which was completed during 2 weekdays and 1 weekend day assigned at random for each individual [16]. All the food and drinks consumed at home and away were recorded in a 3-day diary. Each day of the diary comprised of six meal slots labelled: breakfast, midmorning, lunch, midafternoon, dinner, and “late evening and night.” During the first part of the checkup, the subjects received instructions from a dietitian on the procedures for completing the dietary record and measuring food portions. Detailed guidance notes were provided at the beginning of the diary to assist subjects in describing portion sizes using g, ml, or household measures units. One week later, during the second part of the checkup, with the presence of the subject, the diary was checked, completed, coded, and quantified by the dietitian using colored photographs of foods, each with 3 different portion sizes. Two intermediate and extreme portions could also be chosen, yielding a total of 7 choices for estimating quantities consumed [17]. 

The daily consumption of 18 generic main food groups was computed as the mean value of the 3 days: milk, yogurt and fresh/uncured cheese, cheese, eggs, fish, poultry, meat and organs, pork-butcher's meat, snacks, cereals and pasta, bread and toast, pastries and cookies, sugar and confectionery, pulses, potatoes, other vegetables than potatoes, fruits, and added fats and vegetable oils. By using data of the 3-day diary, total alcohol was calculated as the sum of ethanol in all the types of specific alcoholic beverages and expressed in grams of pure alcohol per day using the French Food Composition Database of INRA [18]. 

Data about lifestyle were collected by using relevant questionnaires [14], including information concerning smoking, and education. Weight and height were measured while the participants were standing in light clothing without shoes. Body mass index (BMI) was calculated as weight (kilogram) divided by height (meter) squared.

### 2.3. Statistical Analysis

Statistical analyses were performed using SAS software (version 9.1; SAS Institute Inc, Cary, NC, USA). Subjects were ranked according to their alcohol consumption in 4 groups: (non-)occasional drinkers (0–2 g/day), 3–22 g/day (<2.0 standard drinks), 23–44 g/day (2 to 3.9 standard drinks), and 45–112 g/day (4 to 10 standard drinks). Intake of 22, 44, and 112 g of alcohol correspond to the consumption of 1/4, 1/2, and 1.3 liter of wine; 1/2, 1, and 2.6 liters of beer; 2, 4, and 10 standard drinks of spirit, respectively. In addition, drinkers (consuming 3–112 g/day) were classified according to their beverage preference in 4 categories (wine preference, beer preference, spirit preference, and no preference). A preference for a specific beverage type was defined as an intake of pure alcohol ≥50% of the total alcohol intake. A drinker with no preference was defined as a person whose intake of none of the specific beverage types exceeded 50%. No individual preferred two beverages equally (50% versus 50%).

Exploratory principle component analysis (PCA) was performed to define dietary patterns by using data from the 18 main food groups [19], followed by orthogonal (varimax) rotation to assist in interpretation of the factors and to ensure that the factors were uncorrelated. PCA aggregates specific food groups on the basis of the degree to which items in the dataset are correlated with one another. All variables in PCA were adjusted for nonalcohol energy intakes. Factors with an eigenvalue greater than 1 were retained. Variables with factor loadings having absolute values of ≥0.20 were used in interpreting the factors. Scores were computed for rotated factors as the sum of products of observed variables multiplied by their factor loading.

ANOVA or Kruskal-Wallis tests were used to compare differences between groups. Firstly, baseline characteristics of the participants were computed and compared according to alcohol intake and alcoholic beverage patterns. Secondly, relationships between alcohol intake and diet were assessed by using ANOVA with the hypothesis of linear trend and after adjustment for age, nonalcohol energy intakes, cigarette smoking, body mass index, education, and season. Thirdly, associations of diet with pattern of alcoholic beverage consumption were tested using ANOVA after adjustment for age, alcohol consumption, nonalcohol energy intake, cigarette smoking, body mass index, education, and season. (Non-)occasional drinkers and drinkers with no preference were excluded from this latter analysis. *P* ≤ 0.05 was accepted as significant.

## 3. Results

The characteristics (especially the covariates used in ANOVA) of the 423 males are shown in Table 1. A positive significant association between increasing alcohol intakes and proportion of smokers and number of cigarettes smoked per day was found. Wine drinkers were older than beer and spirit drinkers and consumed more pure alcohol per day. Spirits drinkers had significant higher body mass index and higher education status.

Two major dietary patterns with eigenvalue greater than 1 were identified by factor analysis using varimax rotation (Table 2). The first factor (eigenvalue = 1.24) was characterized by higher consumption of sugar and confectionery, added fat including vegetal oil, fruits, vegetables, milk, fish, poultry, and eggs and lower consumption of meat and organ, pork-butcher's meat, and pastries and cookies. The second factor (eigenvalue = 1.06) was associated with higher consumption of pastries and cookies, and snacks and lower consumption of pork-butcher's meat, cheese, and bread and toast.

Table 3 presents the consumption of food groups across the 4 categories of alcohol intake. By using adjusted values for age, nonalcohol energy intake, cigarette smoking, body mass index, education and season, we observed a negative significant association between increasing alcohol intakes and the consumption of milk, yogurt and fresh/uncured cheese, sugar and confectionery, pastries and cookies, vegetables and fruit, and a significant positive relationship with cheese, meat and organs, pork-butcher's meat, and potatoes. In line with the above cited data, the first dietary pattern was inversely related to alcohol intakes (*P* = 0.002) and there was a significant association between alcohol intake and the second diet pattern (*P* = 0.011): heavy drinkers having lower value of pattern.

When analyzing daily consumption of specific food groups and diet patterns according to beverage preference (wine, beer, and spirits), after adjustment for age, nonalcohol energy intake, alcohol intakes, cigarette smoking, body mass index, education, and season (data not shown), differences for all food groups were not statistically significant. An exception was the consumption of poultry which was significantly higher in the spirit drinkers group (*P* = 0.006). Likewise, diet patterns were not significantly related to beverage preferences: −0.10 ± 0.78, 0.04 ± 0.97, and 0.12 ± 0.61 (*P* = 0.326) for the first diet pattern and 0.01 ± 0.80, −0.08 ± 1.11, and 0.02 ± 0.62 (*P* = 0.275) for the second; for wine, beer, and spirit drinkers, respectively.

## 4. Discussion

In this study, increased alcohol intake was associated with higher consumption of potatoes and animal products such as meat, meat products, and pork-butcher's meat and low consumption of dairy products such as milk, yogurt, and fresh/uncured cheese, and fruits and vegetables. In addition, pastries and cookies and sugar and confectionaries were less consumed by alcohol drinkers. The significant associations of poorer dietary practice with alcohol consumption were underlined previously in various populations around the world [6–9]. 

In line with our above results, the first dietary pattern was inversely related to alcohol intake. Previous studies using a dietary score based on components representing different aspects of a healthy diet such as the Healthy Eating Index in USA (HEI) [20] or the Diet Quality Index in France [9] showed that as alcohol quantity increased, diet index worsened. When cluster analysis or factor analysis was used to search for dietary patterns in various populations, patterns identified and labeled “alcohol and meat products” [21], “alcohol users” [22], “alcohol and convenience foods” [23], or “convenience food/beer” [24] reflected mainly the aggregation of alcoholic beverages with higher consumption of meat and processed meat and lower consumption of low-fat dairy products, desserts, fruits, and vegetables. 

While levels of alcohol consumption were significantly and inversely associated with dietary quality in our sample of healthy adult men, food consumption according to wine, beer, or spirit preference did not significantly differ (except for poultry; spirits drinkers having the highest consumption). Likewise, the first diet pattern identified by factor analysis was not associated with beverage preference. Despite the growing number of studies in the literature, the question about differences in dietary patterns associated with beer, wine, and spirits is still unresolved. Contrary to our results, in studies conducted in United States, Australia or northern Europe [7, 10, 11, 25–27], wine drinkers tended to report healthier dietary patterns: more fruits, vegetables, grains, fish, olive oil, and fewer red or fried meats, sausage, bacon, and fried potatoes versus other groups of drinkers. In Denmark, by using information on number, type of item, and total charge from transactions in supermarkets, wine buyers made more purchases of healthy food items than people who bought beer [28]. Conversely, in studies conducted in Spain or in Italy, no relevant difference in healthy foods consumption and/or in adherence to Mediterranean diet was shown [13, 29, 30]. The study of Ruidavets et al. [9] on 3 population samples of Northern, North-eastern, and South-western France (MONICA study), showed that wine drinkers had healthier diet compared to other drinkers or abstainers. However, in this last French study, the living area played a significant role in the dieting behaviour and also in alcoholic beverage preferences since all associations became nonsignificant after additional adjustment for this parameter. These results are in agreement with the North-South differences for relationships between beverage behaviours and diet patterns.

Discordance of results between North and South areas may be due to the fact that in a specific population (area), various factors aggregate with drinking habits such as regional culture, socioeconomic status, diet and beverage habits, food and beverage availability, food and beverage purchases, attitude and knowledge about potential effects of wine, and other foods on heath. For instance, in Denmark or in California wine drinkers have a higher level of education and higher income, better psychological functioning, and better subjective health than people who do not drink wine [31–33]. Conversely, in Spain or in Italy, wine is largely consumed by all social classes because it is economically affordable for all [13]. In addition, population interests with food and health may be very different: the Northern European and American populations are more inclined to associate food with health and not with pleasure, conversely to French people [34]. 

The present study has some limitations and strengths. First, among the number of method of dietary assessment reported in the literature, the 3-day dietary record (with the 5-day instrument) is considered as one of the reference methods without recall bias, limiting the accuracy or completeness of the information [35]. Moreover, an experimenter reviewed the diaries with the participants using pictures of dishes in order to estimate quantities and clarified any ambiguities or missing data. However, the 3-day dietary record did not allow taking into account the long term variability in comparison to alternative methods such as Food Frequency Questionnaire (FFQ) that better measure long term diet. Second, although factor analysis takes into account the high intercorrelations of foods within the diet, this approach involved several arbitrary decisions such as the component of food groups, the number of factors extracted, the rotation method, the label of factors. Third, respondents' participation in this study was on a volunteer basis, therefore the study subjects may be more health conscious than the general population. As for other epidemiological studies, misreporting of diet, particularly consumption of foods regarded as “unhealthy,” is a major concern when looking for determinants of food intakes. Moreover, the participants in this study were middle-aged men from a specific area of France (the east) where beer drinkers denote a particular population group. These findings may not generalize to younger/older individuals, women, or individuals living in other regions. Fourth, since important differences between the beverage prefererer's were highlighted (wine drinkers had by far the highest alcohol intake and were oldest; beer drinkers had the lowest BMI, smoked the most, with the lowest education); we adjusted for these covariate in the dietary pattern analyses. Another limitation of our study is the low number men having beer or spirit preferences and consequently the possible lack of statistical power.

To conclude, in our study, there was a linear trend between increasing alcohol intakes and worsening of quality of diet. Conversely, in agreement with other data obtained in the south of Europe, food intakes did not significantly differ according to wine, beer or spirit preference. Taking into account limitations due to the small sample of beer and spirit drinkers, this similarity in dietary patterns according to beverage preferences don't support the hypothesis that the positive cardiovascular effects reported in the literature for wine could be attributable to an overall healthier dietary pattern of wine drinkers in particular in this sample from Eastern France.

## Figures and Tables

**Table 1:** Characteristics of the sample of 423 adult males according to alcohol consumption and beverage preference^a^.

	Alcohol intakes (g/day)					Beverage preference			
	0–2 *N* = 93	3–22 *N* = 168	23–44 *N* = 98	44–112 *N* = 64	*P* value^b^	Wine *N* = 265	Beer *N* = 31	Spirits *N* = 23	*P* value^b^
Alcohol (g/d)	0.6 ± 1.1	12.3 ± 5.7	31.3 ± 6.6	65.5 ± 15.6	—	30.9 ± 22.2	16.8 ± 13.8	9.9 ± 6.6	≤0.001
From wine (%)	—^c^	63.6 ± 37.1	71.6 ± 22.9	76.2 ± 18.1	0.009	81.2 ± 15.8	11.5 ± 17.6	11.9 ± 19.0	—
From beer (%)	—	19.7 ± 29.9	14.9 ± 20.2	10.0 ± 11.8	0.022	9.2 ± 11.9	81.1 ± 20.1	7.1 ± 14.8	—
From spirits (%)	—	16.6 ± 28.9	13.4 ± 14.5	13.7 ± 13.3	0.476	9.5 ± 12.6	7.3 ± 14.7	81.0 ± 20.7	—
Age (y)	41.8 ± 5.8	42.5 ± 4.9	42.6 ± 4.9	42.9 ± 4.7	0.509	42.9 ± 5.0	40.6 ± 3.8	41.6 ± 3.2	0.024
Body mass index (kg/m^2^)	26.0 ± 4.6	25.8 ± 3.2	25.1 ± 3.0	25.2 ± 2.9	0.206	25.5 ± 2.9	24.4 ± 2.7	26.7 ± 3.8	0.021
Cigarette (cig/day)^d^	3.4 ± 8.1	3.8 ± 7.4	5.7 ± 9.9	8.4 ± 12.6	0.002	4.9 ± 8.9	6.9 ± 12.1	5.1 ± 8.7	0.506
Smoking behavior (%)									
Nonsmokers	49.5	38.1	29.6	15.6		32.4	35.5	17.4	
Smokers	18.3	28.0	36.7	42.2	≤0.001	32.5	35.5	30.4	0.413
Ex-smokers	32.2	33.9	33.7	42.2		35.1	29.0	52.2	
Education (%)									
Primary school	57.0	51.8	47.0	51.6		47.5	71.0	52.2	
Secondary school	25.8	25.6	31.6	28.1	0.840	30.6	12.9	13.0	0.036
University	17.2	22.6	21.4	20.3		21.9	16.1	34.8	

^a^Mean ± SD or percent.

^b^ANOVA for continuous variables (except for cigarette smoking) or Chi-square test for categorical variables.

^c^Not relevant, ^d^Kruskal-Wallis test.

**Table 2:** Factor-loading matrix for the major factors (diet pattern) identified by using food group intakes^a^.

	Factor-loading patterns^b^	
	First pattern	Second pattern
Sugar and confectionery	0.449	—^c^
Added fats and vegetable oils	0.395	—
Other vegetables than potatoes	0.287	—
Fruits	0.256	—
Milk	0.253	—
Fish	0.232	—
Poultry	0.230	—
Eggs	0.209	—
Yogurt and fresh/uncured cheese	—	—
Cereals and pasta	—	—
Potatoes	—	—
Pulses	—	—
Meat and organs	−0.397	—
Pork-butcher's meat	−0.418	−0.260
Pastries and cookies	−0.227	0.672
Snacks	—	0.207
Cheese	—	−0.218
Bread and toast	—	−0.592
% of explained variance	24.2%	20.7%

^a^All variables were adjusted for nonalcohol energy intakes

^b^Factor loadings represent the correlations between the variables and the factors.

^c^Factor loading <0.20.

**Table 3:** Daily intake of foods and nutrients according to alcohol consumption in the sample of 423 adult males^a^.

	Alcohol intakes (g/day)				
	0–2 *N* = 93	3–22 *N* = 168	23–44 *N* = 98	45–112 *N* = 64	Adjusted *P* for trend^b,c^
Foods					
Milk (g)	160.2 ± 170.0	111.2 ± 153.6	81.9 ± 108.4	85.3 ± 113.6	≤0.001
Yogurt and fresh/uncured cheese (g)	73.3 ± 88.2	61.0 ± 71.0	40.6 ± 52.1	20.0 ± 40.3	≤0.001
Cheese (g)	52.3 ± 41.4	49.8 ± 32.4	56.4 ± 33.3	61.3 ± 41.3	0.037
Eggs (g)	16.9 ± 21.1	17.6 ± 19.9	18.4 ± 21.1	22.5 ± 24.4	0.152
Fish (g)	37.9 ± 52.2	31.3 ± 35.8	35.0 ± 39.8	26.9 ± 35.7	0.189
Poultry (g)	30.1 ± 40.5	30.9 ± 37.2	25.0 ± 38.9	27.7 ± 31.7	0.491
Meat and organs (g)	79.1 ± 50.1	86.4 ± 55.1	93.0 ± 53.9	105.1 ± 60.2	≤0.001
Pork-butcher's meat (g)	58.5 ± 57.0	65.3 ± 60.9	82.8 ± 65.2	89.3 ± 62.4	≤0.001
Snacks (g)	21.1 ± 42.9	26.2 ± 44.6	25.3 ± 43.8	25.7 ± 37.3	0.246
Cereals and pasta (g)	105.3 ± 98.2	104.2 ± 84.9	89.1 ± 84.9	93.4 ± 66.8	0.199
Bread and toast (g)	168.6 ± 91.3	154.4 ± 84.0	154.8 ± 76.7	159.3 ± 78.4	0.508
Pastries and cookies (g)	68.3 ± 65.8	85.5 ± 79.4	66.8 ± 64.4	47.3 ± 40.6	0.018
Sugar and confectionery (g)	46.0 ± 30.9	38.6 ± 34.8	32.0 ± 30.0	31.5 ± 23.5	≤0.001
Pulses (g)	18.1 ± 37.7	17.5 ± 34.4	13.5 ± 25.7	15.6 ± 27.8	0.351
Potatoes (g)	86.0 ± 76.9	94.1 ± 78.3	106.2 ± 95.8	108.3 ± 92.3	0.027
Other vegetables (g)	226.3 ± 146.5	224.6 ± 126.4	194.3 ± 96.9	174.6 ± 100.5	0.002
Fruits (g)	139.7 ± 128.3	121.0 ± 108.2	102.1 ± 98.3	90.1 ± 99.7	≤0.001
Added fats and vegetable oils (g)	28.1 ± 18.6	27.4 ± 18.8	29.3 ± 18.3	30.2 ± 19.4	0.427
Diet pattern					
First pattern	0.28 ± 0.82	0.04 ± 0.83	−0.16 ± 0.77	−0.25 ± 0.70	0.002
Second pattern	−0.02 ± 0.84	0.17 ± 0.86	−0.08 ± 0.81	−0.30 ± 0.64	0.011

^a^Mean ± SD.

^b^ *P* for linear trend after adjustment for age, nonalcohol energy intakes, cigarette smoking, BMI, education, and season.
